# Electrochemical reduction of CO_2_ to ethylene on Cu/Cu_*x*_O-GO composites in aqueous solution†

**DOI:** 10.1039/d0ra02754e

**Published:** 2020-05-06

**Authors:** Nusrat Rashid, Mohsin Ahmad Bhat, U. K. Goutam, Pravin Popinand Ingole

**Affiliations:** Indian Institute of Technology Delhi 110016 India ppingole@chemistry.iitd.ac.in; University of Kashmir Srinagar 190006 India; Raja Ramanna Centre for Advanced Technology Indore 452013 India

## Abstract

Here, we present fabrication of Graphene oxide (GO) supported Cu/Cu_*x*_O nano-electrodeposits which can efficiently and selectively electroreduce CO_2_ into ethylene with a faradaic efficiency (F.E) of 34% and a conversion rate of 194 mmol g^−1^ h^−1^ at −0.985 V *vs.* RHE. The effect of catalyst morphology, working electrode fabricational techniques, the extent of metal–GO interaction and the oxide content in Cu/Cu_*x*_O, was studied in detail so as to develop a protocol for the fabrication of an active, stable and selective catalyst for efficient electro-production of ethylene from CO_2_. Moreover, a detailed comparative study about the effect of the GO support, and the nature of the cathodic collection substrate used for the electro-deposition is presented.

## Abstract 2

Herein, we present fabrication of graphene oxide supported Cu/Cu*_x_*O nano-electrodeposits which efficiently and selectively can electroreduce CO_2_ into ethylene with a faradaic efficiency of 34% and conversion rate of 194 mmol g^−1^ h^−1^ at −0.985 V *vs.* RHE.

## Graphical abstract

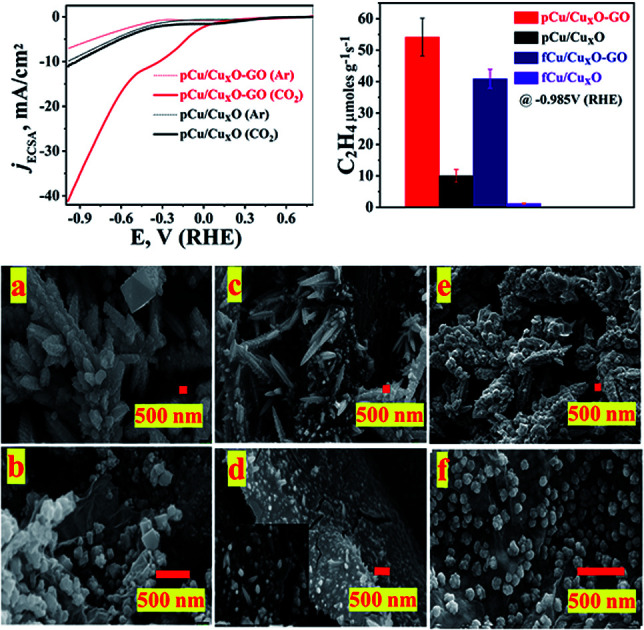

## Introduction

The amount of atmospheric carbon dioxide is continuously increasing, for example, CO_2_ concentration in the atmosphere was recorded at its highest in May 2019, with the average at 414.7 parts per million (ppm) at NOAA's Mauna Loa Atmospheric Baseline Observatory. In response, intense research activity has been devoted globally to not only deal with carbon emissions but also to utilize CO_2_ as a resource for beneficial processes through the development of economically viable technologies for environmental remediation. In this regard, the electrochemical reduction of CO_2_ (ERC) has attracted considerable attention to address the problem of global climate change.^1–4^ In particular, conversion of CO_2_ to hydrocarbons like methane,^5–7^ ethane, ethylene, *etc.* which are energy dense fuels and chemicals, would be an added advantage.

However, due to higher overpotentials, less selectivity and low stability, the ERC needs catalysts capable of selectively reducing CO_2_ at less overpotentials and with long run times.^8,9^ Various copper-based materials have been tested for electrochemical reduction of CO_2_ into hydrocarbons, and it has been observed that the morphology and surface area of catalyst directly affects the overall activity of the ERC.^10–12^ However, due to the low stability of copper and copper oxide for ERC, the use of support materials like carbon nanostructures have been employed both to enhance the selectivity and to increase the robustness of the catalyst.^13,14^ Furthermore, the support materials also plays a role in determining the selectivity as well as total efficiency in reducing CO_2_.^15^

Overall, the reported studies suggest that the optimization and exploitation of the following aspects would be important toward further improvements in ERC activity of Cu based electrocatalysts; (i) altering the surface structure (morphology) of Cu particles to increase the number of surface active sites, this may also include the porous nature of the surface (ii) fine tuning of the relative extent of metallic Cu to the oxide contents on Cu surface, and (iii) selection of an appropriate support material for the Cu deposits. In this regard, design and development of ERC catalyst based on Cu-nanostructures with optimum oxide content and an appropriate support material is an important step towards effective and selective conversion of CO_2_ into hydrocarbons. Among the various supports, graphene and its related forms such as graphene oxide (GO) have been extensively used for their advantageous physico-chemical properties including^16^ high surface area, good electronic conduction characteristics and easy to functionalize structure. We along with other researchers have reported the successful immobilization of noble metallic nanoparticles on graphene/GO sheets that significantly enhance the catalytic and sensing activities of these nano-composites as the GO supports acts as both electron acceptor as well as donor.^17–20^ The question is how well the GO as support can be utilized to control the product of ERC generated over Cu-NPs in contact with the graphene support. Hence, in order to understand the effect of Cu-nanoparticles supported on GO sheets for electrochemical conversion of CO_2_ into hydrocarbons is important to probe. Motivated with these properties of GO, we successfully attempted the synthesis of Cu/Cu_*x*_O-GO composite and its utilization for electro-catalytic activity towards CO_2_ reduction. Furthermore, we studied the effect of three different aspects of the catalysts *viz.* catalyst morphology, the extent of metal–GO interaction and the oxide content in the Cu/Cu_*x*_O on the electro-catalytic activity towards ERC. Furthermore, an insight into the effect of fabrication techniques for both catalyst and working electrode has been attempted.

For this, a simple and scalable electrochemical deposition approach was used to fabricate the different electrodes based on Cu/Cu_*x*_O and Cu/Cu_*x*_O supported on GO sheets under varying conditions. For example, pCu/Cu_*x*_O, a powdered sample acquired after drying of electrodeposits (obtained with Pt-plate cathode) was mixed with isopropanol and water mixture to make a catalyst ink which was then pressure sprayed on carbon fiber paper (CFP) to obtain pCu/Cu_*x*_O modified CFP electrodes. Similarly, a catalyst ink of pCu/Cu_*x*_O-GO, a powdered sample synthesized with Pt plate as a cathode but in presence of GO suspension was pressure sprayed on CFP to get pCu/Cu_*x*_O-GO modified electrode. For fabricating fCu/Cu_*x*_O and fCu/Cu_*x*_O-GO, the respective samples were directly electrodeposited on CFP from their electrodeposition baths, using CFP as a cathode (*i.e.* without removing the electrodeposit from CFP surface). For the comparison of mode of electrode fabrication, a few electrodes were also fabricated in a layer-by-layer fashion. Thus, overall, we have fabricated six different electrodes and comparatively studied their catalytic activity towards ERC. Among the studied samples, pCu/Cu_*x*_O-GO nanocomposite shows the best activity with an overall faradaic efficiency of *ca.* 72% at an applied potential of −0.985 V *vs.* RHE with ethylene production at a rate of 194.4 mmol g^−1^ h^−1^ or 34% of F.E.

## Experimental section

### Materials

All the chemicals used for the synthesis and electrochemical measurements were of analytical reagent grade and were used as such without further purification unless otherwise mentioned. Trisodium citrate (TSC) (99%) was procured from Merck India, Limited. Graphene oxide dispersion in water (4 mg mL^−1^) and potassium hydrogen carbonate (KHCO_3_) (99.9%) were purchased from Sigma Aldrich. Ultrapure CO_2_ and N_2_ gases were procured from Sigma Gases, New Delhi and before using for electrochemical measurements were passed through a filter equipped with an oxygen trap, gas purifier and moisture trap to remove the traces of impurities (Prama Instruments Pvt. Ltd.). Copper metal sheet (99.9% pure, metal basis) was purchased from Alfa Aesar. Carbon fibre paper (CFP) sheets (TGPH-120) were procured from Nickunj Exim Entp. Pvt. Ltd. India.

### Synthesis of Cu/Cu_*x*_O-GO composites in powder form and thin films

The Cu/Cu_*x*_O-GO composites were prepared in two different forms, powder and thin films by using a DC electrophoretic anodization of metallic copper foil in presence of GO suspension in aqueous tri-sodium citrate solution with slight modification in our previously reported recipe.^21^

#### Powder form

Typically, for the synthesis of Cu/Cu_*x*_O and its composites, a metallic copper foil (2 cm × 2 cm) mechanically polished with a smooth polishing paper followed by electrochemical polishing in 85% phosphoric acid, was used as a sacrificial anode. Pt plate (2 cm × 2 cm) cleaned in distilled water by ultra-sonication for 5 minutes was used as a collector (*i.e.* for electrodeposition) electrode/cathode. A positive load of 15 V was applied (Keithley 2231A-30-3 triple channel DC power supply) for 60 minutes at a fixed temperature of 40 °C (±1 °C) in an electrolytic bath containing 200 mM tri-sodium citrate aqueous solution with electrolyte contents stirring continuously.

After 60 minutes the electrodeposits were tapped off from platinum foil and collected in centrifuge tubes, washed multiple times with copious amount of water followed by ethanol and finally by acetone before drying it under vacuum for 6 hours. The powder obtained as such was named as pCu/Cu_*x*_O (where p represents samples obtained in powder form at Pt plate cathode). To synthesize the composite, 0.3 mg mL^−1^ GO was added to the 200 mM aqueous tri-sodium citrate solution with all other synthesis parameters like applied voltage, temperature of bath, concentration of tri-sodium citrate, and anode and cathode kept unaltered, the sample obtained was named as pCu/Cu_*x*_O-GO (powder sample obtained with Pt as cathode and GO as a support material). These materials were then pressure sprayed on CFP for fabricating electrode for studying ERC.

#### Thin films

For the fabrication of films, a similar experimental setup was used except that the films were now directly electrodeposited on CFP in place of Pt for three minutes. Thus, obtained modified CFP was rinsed with water and dried under the flow of nitrogen gas, and named as fCu/Cu_*x*_O (where f signifies the materials were obtained as films directly on CFP). To synthesize fCu/Cu_*x*_O-GO (film directly deposited at CFP with GO as support material) composite, similar electro-deposition was carried out but in presence of GO suspension (0.3 mg mL^−1^) in otherwise a replica of other synthesis parameters. Further to check the effect of step-by-step electro-deposition, a CFP was first modified with GO (by pressure spraying) and then Cu/Cu_*x*_O was electrodeposited on it, and the electrode is named as fCu/Cu_*x*_O@GO (a thin film of Cu/Cu_*x*_O electrodeposited on a layer of GO which was pressure sprayed on a CFP).

### Material characterization

The solid-state properties of materials were analysed by X-ray diffractometer (XRD) Bruker model D8 Advance with Cu Kα (*λ* = 1.54 Å) and a scan speed of 0.2 seconds/increment with 2*θ* range of 10° to 60°. A reference method was used to record XRD patterns of the materials where an equal amount of sample was spray dried on CFP for the powdered samples, and thin film samples were used as such for the measurements. The field emission scanning electron microscope (FESEM) attached with Oxford-EDS system IE 250X max 80 with latest 80 mm2SDD detector model FEI Quanta 200 was used to image the morphology and elemental composition of the prepared materials. Elemental mapping was done on Hitachi High technol system. Renishaw plc micro Raman spectrometer under 514 nm laser was used to get the characteristics of the composites. For this, a certain amount of powder was scraped off from the CFP and pressed into a pellet. X-ray photoelectron spectroscopy (XPS) measurements were carried using synchrotron radiation facility hard X-ray photoelectron spectroscopy (HAXPES) beamline BL-14, Indus-2 which has double-crystal monochromator [Si (111)] with excitation energy of 4.311 keV and equipped with Hemispherical analyser and detector system (Phoibos 225, Specs make). The typical pressure in the experimental station is 5 × 10^−9^ mbar. Survey spectra were recorded over a range of 0 to 1000 eV for each composition. The energy calibration has been done with respect to the reference (C 1s peak at a binding energy value of 284.8 eV adventitious carbon) for all edges.

### Electrochemical measurements

All the electrochemical measurements were carried out on Metrohm Autolab PGSTAT302N with standard three electrode system in a custom designed two compartment cell with a Nafion 117 (DuPont) membrane. The CFPs modified with Cu/Cu_*x*_O or Cu/Cu_*x*_O-GO has been used as working electrode, Ag/AgCl (saturated with KCl) as a reference electrode in cathode chamber and Pt foil as counter electrode (separately) in an anode chamber. All the potentials were referenced *vis-a-vis* reversible hydrogen electrode using the formula *E*_RHE_, V = *E*_(Ag/AgCl)_, V + 0.197 V + (0.059 × pH) V after correcting for uncompensated ohmic losses determined through impedance measurements. 0.2 M KHCO_3_ saturated with either argon or CO_2_ was used as an electrolyte medium for all the CO_2_ reduction measurements, unless mentioned otherwise. 0.1 M HClO_4_ was used to record the CVs (cyclic voltammograms) for electrochemical surface area (ECSA) measurements. In order to avoid the effect of pH variation in CO_2_ purged solutions (generally, pH decreases upon CO_2_ purging as solution becomes more acidic), pH of the argon saturated electrolyte solutions was adjusted to 7.00 which was the pH of solutions saturated with CO_2_ (purging for an hour with 20 mL min^−1^ of flow rate).

### Preparation of electrodes

Working electrodes for ERC were fabricated in following ways; (i) electrodeposited thin films (fCu/Cu_*x*_O, fCu/Cu_*x*_O-GO and fCu/Cu_*x*_O@GO) on CFP cathode were used as such without any modifications, (ii) for samples obtained as powder, 2.5 mg of each was dispersed in 3 : 1 iso-propanol and water mixture, with 5 μL Nafion for 30 minutes and the dispersion was fed to an air spray gun and coated as a film on the CFP (2 cm × 1 cm) under the flow of nitrogen as carrier gas. To understand whether direct electro-deposition or mechanical fabrication of electrodes – catalyst manually painted on the electrode is better, few more electrodes were fabricated in the following manner. (iii) CFP was modified with GO by pressure spray, and further air sprayed with Cu/Cu_*x*_O and was labelled as pCu/Cu_*x*_O@GO. (v) Finally, to understand the synergism between Cu/Cu_*x*_O and GO, CFP electrode was modified with only GO and was named as GO. All care was taken to mask the rest of the electrode surface so that for all the fabricated electrodes identical area of CFP cathode is exposed for equal geometric surface area, and also similar sample loading was kept by calculating mass deposited in case of films by the amount of charge passed. The gaseous products obtained in electro-reduction of CO_2_ were analysed by Thermo Fischer Trace 1100 gas chromatograph with TCD and FID analysers for detection of hydrogen and other ERC products respectively after passing through a Carboxene column at a ramping temperature of 50 °C to 240 °C.

Liquid products were analysed with Bruker Advance AV-III (400 MHz) NMR by diluting 0.5 mL catholyte with 0.1 mL D_2_O (as NMR solvent) and 0.5 μL di-methylsulphoxide (DMSO)-as internal standard. The solvent suppression method with a pre-saturation pulse was used to cater the overwhelming solvent peak, which otherwise renders the ECR product peaks as unobservable.

### Calculation of faradaic efficiency

The faradaic efficiency (F.E.) of the collected gaseous products was calculated using eqn (1),1
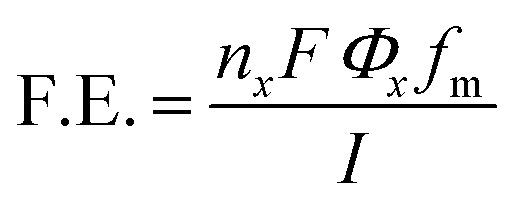
where, *n*_*x*_ is number of electrons involved in generation of product under consideration (*e.g. n*_*x*_ = 2 for CO; 8 for CH_4_ and 12 for C_2_H_4_), *F* is Faraday's constant (96 485 C mol^−1^), *Φ*_*x*_ is the volume fraction of the gaseous products (considering gases to be ideal), *f*_m_ is the molar flow rate (mol s^−1^), and *I* is the corresponding current (in ampere, A). Sierra Instruments, USA Make Smart-Track 100 Series Digital Mass Flow Controller (C100L-DD-2-OV1-SV1-PV2-V1-S0) with Built-in 10 gas calibration system was used for controlling and measuring the flow rate of CO_2_ purging. Faradaic efficiency of liquid products was calculated by method mentioned elsewhere.^22^

For liquid products, the *R* factor was calculated as given below2
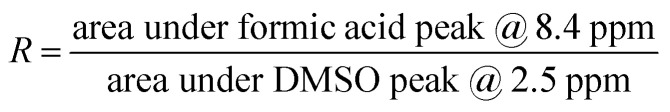
*R* for different concentrations of formic acid was calculated, and a calibration curve was plotted with *R vs.* concentration of formic acid. The slope of the *R vs.* concentration of formic acid was further used for calculation of faradaic efficiency for formic acid production which is the primary reaction product in liquid form.Formic acid conc. = *R*/slope, M^−1^

The total catholyte amounted to 50 mL in cathodic chamber; therefore, the total concentration of formate produced was,*N*_formic acid_ = 0.05 L × formic acid concentration.

Number of moles of electrons used to produce n moles of formic acid are*e*_output_ = *N*_formic acid_ × 2e^−^

Total number of moles of electrons applied during the time of electrolysis was*e*_input_ = *Q*/*F*where *Q* is the charge passed and *F* is the Faraday's constant. The F.E. was calculated by,
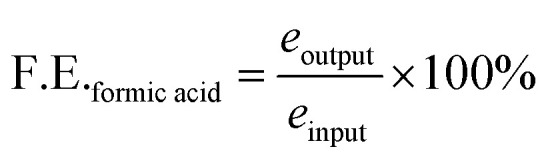


## Results and discussion

**Fig. 1:**
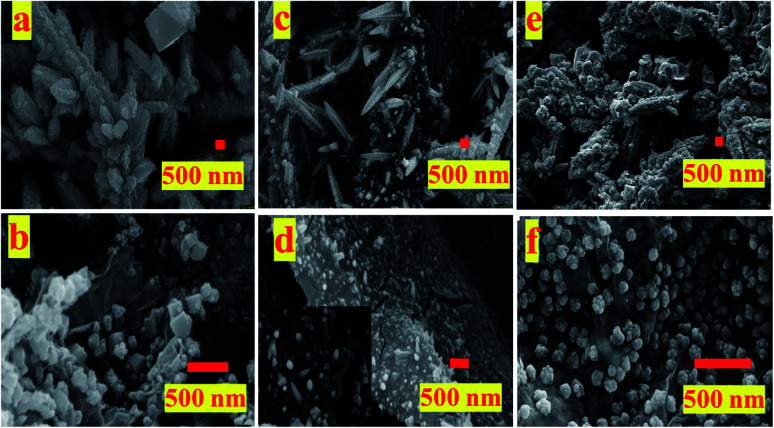
FESEM images of different materials used as electrodes for ERC; (a) pCu/Cu_*x*_O, (b) pCu/Cu_*x*_O-GO, (c) fCu/Cu_*x*_O, (d) fCu/Cu_*x*_O-GO, (e) pCu/Cu_*x*_O@GO (physical layer over layer composite), (f) fCu/Cu_*x*_O@GO – film of Cu/Cu_*x*_O electrodeposited on GO layer.

The FESEM images of the samples (Fig. 1) shows self-assembly of these catalysts depends upon the substrate used for electro-deposition and whether or not GO was present in the electrolytic bath. While in absence of GO, the obtained Cu/Cu_*x*_O particles are self-assembled into either free cones or standing dendrimers, the presence of GO restricts their growth to small particles adhered to its surface. GO in solution because of its electrical properties alters the solution conductivity which may affect both mass transport as well as co-deposition of the copper nanostructures. Effect of GO on synthesis of these catalyst is elucidated in Fig. SI-1.† Fig. SI-1† presents the electrochemical impedance spectroscopy (EIS) graphs obtained in reaction bath with and without GO with Pt plate or CFP as working electrode at open circuit potential (OCP). It is inferred from the graph that there is a slight change in the conductivity of the solution with Pt being more conductive as compared to CFP. The same observation can be seen for presence of GO, the conductivity of solution increases in presence of GO, owing to the electronic properties of the GO. The decrease in solution resistance in GO containing electrolyte is observed irrespective of the working electrode used (Pt or CFP). This observation suggests that GO not only plays a role in the growth of Cu/CuxO but also effects the mass transport of the Cu cations towards the cathode, hence guides the nano-morphology. Similarly, other inference from the EIS data is that the cathode or collection substrate plays a role in growth of the nanostructures. This observation indicates that not only a carbonaceous support but also the electrical properties of cathode play a crucial role in formation of a catalyst. Lower resistance with Pt cathode as compared to CFP cathode, in the presence of GO, may dictate the interaction within the GO framework and the deposited Cu/Cu_*x*_O NPs, though this needs further detailed investigation.

From the FESEM images (Fig. 1), it is observable that the presence of GO has limited the growth of Cu/Cu_*x*_O composites (pCu/Cu_*x*_O-GO (Fig. 1(b))) and fCu/Cu_*x*_O-GO (Fig. 1(d)) along the (100) growth axis thus producing either cubes or small finger-like spherical protrusions adhered to the surface of CFP. The electrodeposited Cu/Cu_*x*_O on the GO layer hand-painted on CFP (fCu/Cu_*x*_O@GO) shows the presence of Cu/Cu_*x*_O spherical particles on GO surface. The physical layer-over-layer (Cu/Cu_*x*_O over GO) *i.e.* pCu/Cu_*x*_O@GO (Fig. 1(e)) shows structures like pCu/Cu_*x*_O on GO sheets. For the characterization of crystalline features of the prepared catalysts, X-ray diffraction (XRD) patterns (Fig. SI-2†) were recorded on the obtained materials which suggest the formation of Cu and Cu_*x*_O mixed phases in all the samples. However, the collection substrate (cathode used in electrodeposition) shows profound effect on the extent of oxide content. Moreover, Raman spectroscopy data (Fig. SI-3†) support the formation of a composite and deduce an idea about the extent of reduction of GO into rGO (reduced graphene oxide because of composite formation) (which also reflects the extent of defects in GO sheets manifested in *I*_D_/*I*_G_ ratio) hence a presentation of extent of metal–GO interactions.

**Fig. 2:**
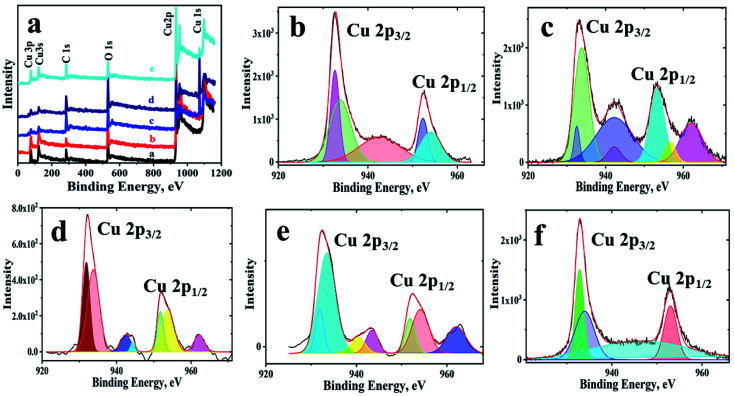
XPS spectra of the Cu/Cu_*x*_O-GO composites (a) survey spectrum of the materials; and detailed spectra of Cu 2p, (b) pCu/Cu_*x*_O, (c) pCu/Cu_*x*_O-GO, (d) fCu/Cu_*x*_O, (e) fCu/Cu_*x*_O-GO, and (f) fCu/Cu_*x*_O@GO.

Further, elemental mapping as well as EDX have been used to demonstrate the distribution of the elements over the observed structures. The colour maps and EDX shows (Fig. SI-4†) the presence of copper, oxygen and carbon with a homogenous distribution and free of any other impurities. The XPS survey spectrum (Fig. 2(a)) of the materials shows peaks for carbon, oxygen and copper only, indicating no other elements of any nature are present. Table SI-2† lists the peak positions of the copper, oxygen and carbon in different materials along with the ratio of Cu^2+^ to Cu^+^. In all of the samples, Cu(ii) state is predominant, and its content is more in composites. From the C 1s and O 1s detailed spectrum shown in Fig. SI-5 and SI-6,† it can be concluded that oxygen functionalities of GO are used by copper leaving more of sp^2^ hybridized carbon backbone in GO. Another interesting observation in XPS is in the case of fCu/Cu_*x*_O@GO, which has an underlying GO layer. The charge transfer between GO and copper has not taken place effectively and hence has given rise to –C–O and –COOH peaks as observed in copper oxides without GO. The peak position of Cu(i) including Cu (0) at 932.6 ± 0.1 eV in powdered Cu/Cu_*x*_O-GO composites can be seen, and peaks of Cu(ii) at 933.8 ± 0.2 eV are also evident along with the associated satellite peaks. The range of energies and the various binding energies are in good agreement with literature reports.^23–25^

**Fig. 3:**
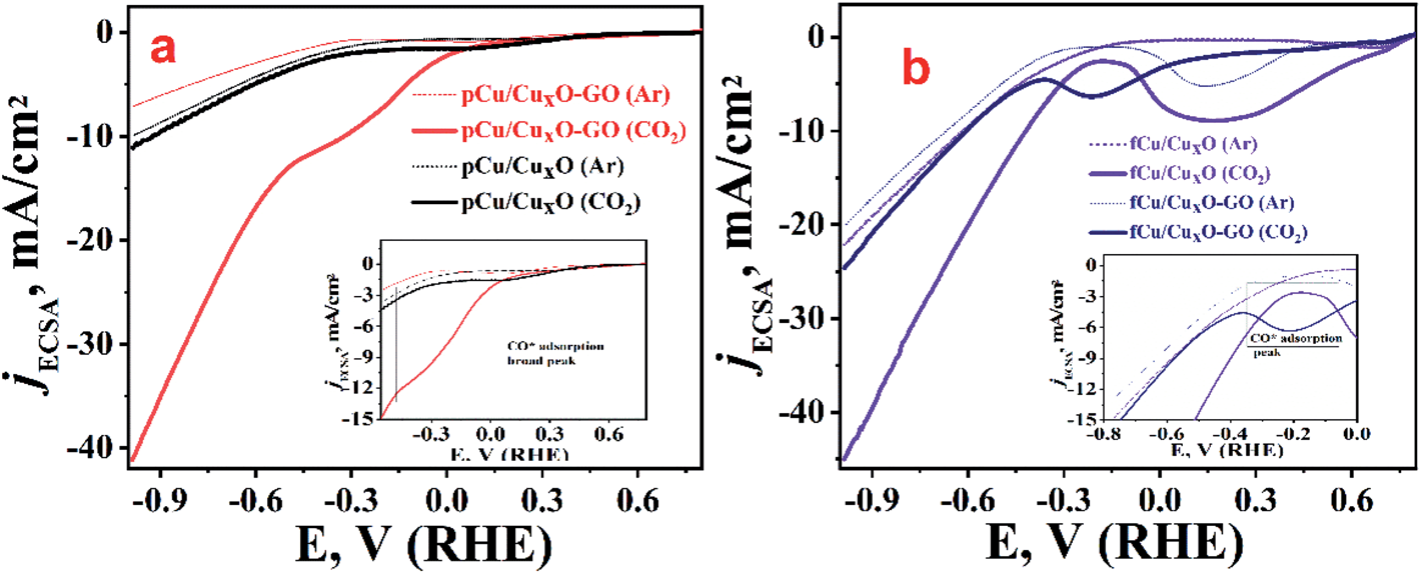
LSV curves in 0.2 M KHCO_3_ electrolyte argon saturated (dashed lines) and CO_2_ saturated (solid lines); (a) pCu/Cu_*x*_O (black traces) and pCu/Cu_*x*_O-GO (red traces), (b) thin films; fCu/Cu_*x*_O (violet traces) and fCu/Cu_*x*_O-GO (navy trace). The enlarged portions of the respective plots are shown as an inset in the respective figures.

To study the electro-catalytic activity of these samples towards, ERC, cathodic linear sweep voltammetry (LSV) curves were recorded for all materials both in presence as well as in the absence of CO_2_. Fig. 3 depicts the overlay of the linear polarization curves normalized with their respective electrochemical surface area (ECSA) (the details of the ECSA measurements which was done using capacitance measurements are given in Fig. SI-7†). Solid lines in Fig. 3 represent the currents observed in presence while dashed ones corresponds to LSVs recorded in absence of CO_2_ (but in presence of argon). In presence of argon, the LSVs shows lower current than ECR along with higher onset potential before reasonable current is achieved. This current at higher potential obtained with argon-saturated 0.2 M KHCO_3_ electrolyte can be attributed to the reduction of water which was also corroborated by gases collected from bulk electrolysis where none of the CO_2_ reduction products other than hydrogen was found. When solution was saturated with CO_2_ and LSV recorded there was a shift in the onset potential towards lower negative potentials along with increased current density which can be attributed to the electro-reduction of CO_2_.

**Fig. 4:**
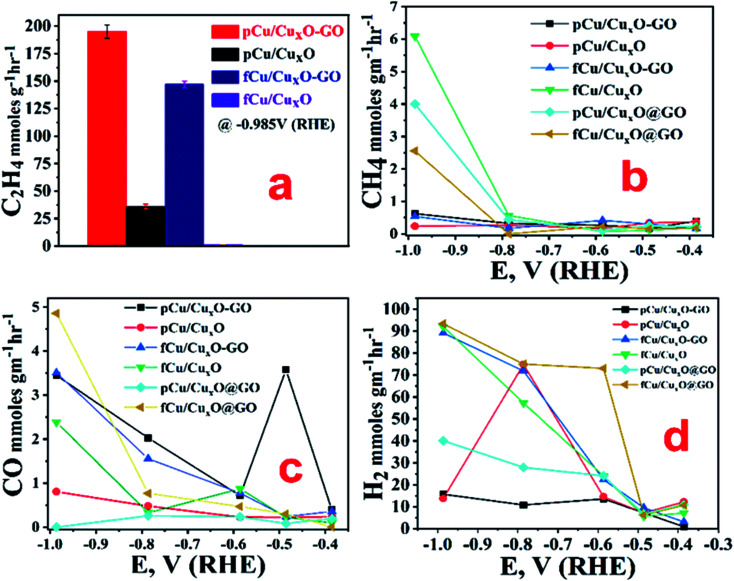
Production rates of ECR products on different catalysts in CO_2_-saturated 0.2 M KHCO_3_ under continuous CO_2_-purging for an hour. (a) C_2_H_4_ at −0.985 V *vs.* RHE (error bars are calculated from standard deviation of three consecutive records), and (b) CH_4_, (c) CO, and (d) H_2_ at different applied potentials.

From the measurements of currents at −0.985 V (RHE) we can see that maximum reduction current is obtained with fCu/Cu_*x*_O (*i.e.* 45 mA cm^−2^) followed by the pCu/Cu_*x*_O-GO (*i.e.* 42 mA cm^−2^) in the presence of CO_2_. However, from the electrolysis product analysis (Fig. 4) done using gas chromatography and NMR measurements on gaseous and liquid products, respectively, it has been observed that pCu/Cu_*x*_O-GO catalyst leads to the hydrocarbons as ECR products whereas the current on fCu/Cu_*x*_O catalyst is due to mostly H_2_ evolution. Although, the electrodes modified with fCu/Cu_*x*_O-GO show the current densities of 25 mA cm^−2^ in presence of CO_2_ the reaction products obtained was majorly H_2_ gas. The electrodes fabricated by layering of catalyst samples are found to be less active for the CO_2_ reduction despite showing higher reduction currents pertaining to more of HER (Fig. 4(d)). Therefore, based on these observations, we found that pCu/Cu_*x*_O-GO catalyst is the most active towards conversion of CO_2_ into hydrocarbons especially selectively into ethylene. Further detailed analysis of the electrolysis products, *viz.* methane, CO, and H_2_ at different applied potentials on different samples is given in Fig. 4. It is pertinent mention here that it is a well-known and reported fact that CO_2_ saturation makes the aqueous electrolyte solutions more acidic. It is also well known that hydrogen evolution becomes more prevalent in acidic solutions^19,26,27^ Hence, the production of H_2_ gas is increased in presence of CO_2_ as compared to that in presence of Ar. However, the competitive production H_2_ can be suppressed over the certain electro-catalyst surfaces that are less active for hydrogen evolution but highly active for ECR. Thus, the larger current density observed for the samples, fCu/Cu_*x*_O and fCu/Cu_*x*_O-GO in presence of CO_2_ is attributed to the lower local (more acidic) pH at the electro-catalyst surface. However, relatively lower hydrogen evolution and higher ERC activity over pCu/Cu_*x*_O.Go and pCu/Cu_*x*_O is attributed to higher selective ERC catalytic activity of these electro-catalysts compared to hydrogen evolution.

It was observed that at very low overpotentials (*i.e.* −0.4 to −0.7 V *vs.* RHE), CO and CH_4_ were detectable but around −0.8 V *vs.* RHE ethylene was also detected in composites but not at Cu_*x*_O without GO. At −0.985 V *vs.* RHE ethylene was the main product with a rate of 194.44 mmol g^−1^ h^−1^ with 34% F.E. on pCu/Cu_*x*_O-GO. Formic acid was the only liquid product detected that too with a very low F.E. of *ca.* 4%. The production of ethylene is probably because of *CO coupling^28^ as can be noticed from the lowered CO production in pCu/Cu_*x*_O-GO composites. It has been documented in the literature that adsorbed CO shows a shoulder or mild peak before ECR and larger the CO adsorption broader the shoulder.^22,29^ Similar observations are noted in the present case as well, where pCu/Cu_*x*_O-GO (Fig. 3(a)) depicts a broad peak for the CO adsorption from 0.3 V to −0.6 V or in some cases up to −0.9 V; hence the presence of ethylene in the ERC product can be attributed to the *CO adsorption followed by dimerization. Importantly, similar broader shoulders are observed for fCu/Cu_*x*_O-GO but not for other materials like fCu/Cu_*x*_O, fCu/Cu_*x*_O@GO and pCu/Cu_*x*_O@GO where the production of ethylene is either zero or very low. For pCu/Cu_*x*_O-GO, the production of hydrogen is also decreased at higher negative potentials (Fig. 4(d)) to a mere F.E. of 16% which can be attributed to the production of more ethylene and increase in local pH. GO showed no appreciable CO_2_ reduction at lower potentials and barely a F.E. of 20% CO production at −0.98 V (RHE) with lower reduction currents.

Fig. SI-8† shows the variation of total current density and F.E. values with time for the ethylene production over pCu/Cu_*x*_O.GO electrode at an applied potential of −0.98 V *vs.* RHE. As can be noted from Fig. SI-8,† the total current density decreases from initial values of −43 mA cm^−2^ to −39 mA cm^−2^ after 10 minutes but then remains constant for an hour long duration. Similarly, the F.E. values for ethylene production remains almost constant over the studied duration of CO_2_ electrolysis.

The obtained trend in the activity of the prepared electro-catalysts can be explained on the basis of the governing physico–chemical properties of these catalyst samples. For example, the deposition substrate employed in an electrophoretic deposition is found to play an important role as it guides the self-assembly of nanostructures and hence the morphology of the resultant electrodeposits. The comparison of the LSV polarization curves for pCu/Cu_*x*_O, fCu/Cu_*x*_O, pCu/Cu_*x*_O-GO, and fCu/Cu_*x*_O-GO (Fig. 3) in CO_2_-saturated electrolyte solutions shows that the catalysts obtained with Pt as a collection substrate depict better activity towards ERC than compared to CPF substrate. This could be due to the loose and porous nature of the materials obtained with Pt plate substrate (Fig. 1) which in turn could be due to the higher hydrogen co-deposition on Pt-plate during electro-deposition of the samples compared to CFP substrate. This has been well established in the literature^30–32^ that higher evolution of gases on the collection substrate leads to higher porosity of the obtained electrodeposits. It is also documented in the literature^33–37^ that the highly porous catalysts provide the higher stability to the intermediates or adsorbed CO giving enough time for C–C coupling on its surface, leading to an increased hydrocarbon production. Thus, the higher yield of hydrocarbons especially ethylene on pCu/Cu_*x*_O-GO can be attributed to the loose and porous nature of these electrodeposits that in turn is an aggregate function of the presence of GO and the nature of cathode material used to collect the electrodeposits. Besides, it can be noted from Fig. 1 that pCu/Cu_*x*_O-GO have asymmetric and cone type shapes and thus higher roughness factor (also observed from ECSA) which increases the total faradaic efficiency (72%) for CO_2_ conversion.

While doing the preliminary experiments, we noticed that the strategy employed for electrode fabrication has a profound effect on the ERC. In general, several researchers, including us, have preferred a layer by layer coating of electro-catalyst over the support material.^38,39^ In contrast, few others have preferred a mixed form of the electrocatalyst and a support material^40^ for studying the electro-catalysis. In order to study the effect of electrode fabrication strategy, electrodes fabricated in three different ways *viz.* fCu/Cu_*x*_O@GO, pCu/Cu_*x*_O@GO and fCu/Cu_*x*_O-GO were investigated for their comparative activities. We found that the electrode obtained with electrochemically co-deposited composites show better activity than the layered one. This comparison is also featured in their physical and electrical properties like XPS and XRD where co-deposited samples were found to yield more interconnected materials which act synergistically to enhance the production of ethylene. Furthermore, it was also noted that simple physical layering of catalyst over GO support are less active than one where copper has been electrochemically anchored to GO.

GO-support has been reported to enhance the catalytic activity and durability of metal and metal oxide NPs towards ERC^41^ as GO not only acts as a conducting scaffold for the catalyst but also increases the robustness of the material. It is well established that during the formation of such hybrids, GO undergoes reduction into reduced graphene oxide (rGO) through charge transfer processes^14,41^ leading to the defect formation in GO, manifested in the ratio of the intensity of D band (*I*_D_) to G band (*I*_G_), *i.e. I*_D_/*I*_G_ ratio. These defects can be decorated with metal catalysts which causes a synergism in transport and conduction of the charges.^42^ We also found the similar enhancement in the ERC activity due to the presence of GO supports in the catalysts. Moreover, we noticed that the catalyst activities are affected by the extent of interaction between GO and copper oxide nanostructures which is manifested in variation of *I*_D_/*I*_G_ ratio. pCu/Cu_*x*_O-GO with highest *I*_D_/*I*_G_ ratio (1.31) showed highest ethylene production followed by fCu/Cu_*x*_O-GO (1.01) and fCu/Cu_*x*_O@GO (0.93). Again, the higher yield of hydrocarbon generation in pCu/Cu_*x*_O-GO with higher degree of defects can be explained on the basis of the higher stability to the intermediates or adsorbed CO giving enough time for C–C coupling on its surface.

Another substantial rationalization that explains the observed trend of activity is the presence of oxide lattices in the synthesized composites. Kanan *et al.*^43^ have reported that the presence of Cu_2_O layers on Cu foil electrode with a higher thickness of ≥3 μm results in lowering of overpotential by 0.5 V than that of polycrystalline Cu. This lowering is mainly due to enhancement in the roughness factor of the electrode surface. While Lee *et al.*^44^ and Mistry *et al.*^45^ claimed residual Cu^+^ ions even after ERC lead to higher ERC efficiencies by using *in situ* XPS or X-ray Absorption near Edge Spectroscopy (XANES) measurements. Other reports by using *in situ* XPS by Eilert *et al.*^46^ suggested the presence of sub-surface oxide layers during ERC changes the electronic structure near Cu, thus increasing the efficiency of ERC. The presence of oxides, along with metallic structures, offer sites with multiple vacancies and increases the catalytic activity of the nanostructures towards ERC.^47^ However, whether such an enhancement is merely due to the roughness factor or the oxygen content has to play any role is not clearly understood or well documented in the literature. This seems to be very important, given the fact that the surfaces of nanostructured materials play a critical role in their physico-chemical as well as electro-catalytic properties. Therefore, in order to have a better insight regarding the effect of oxide content in nano-dimensional copper based electro-catalysts, we tested the pCu/Cu_*x*_O-GO nano-composites with varying O/Cu ratios for their electro-catalytic activity towards ERC. Interestingly, we have noted that the inclusion of oxide form of Cu increases the efficiency of the catalyst in comparison to metallic Cu-rich catalyst for ERC. Table SI-2† shows Cu^2+^/Cu^+^ ratio obtained from XPS of the samples. It can be seen that samples with GO show more of oxide content which could also assist in increasing the efficiency of the composites.^43^

Electrochemical impedance spectroscopy also showed that charge transfer resistance offered by pCu/Cu_*x*_O-GO both at onset as well as at OCP is minimum for ERC among the studied catalysts, other details for EIS are given in ESI (Fig. SI-9, SI-10a and b).† From the trends observed in current study, pCu/Cu_*x*_O-GO was found to be the most active and selective catalyst for CO_2_ conversion into ethylene with 34% F.E. at −0.985 V (RHE). The observed catalytic efficiency appears to be a cumulative effect of change of neighbouring electronic structure of copper nanostructures on GO support,^48^ higher electrochemical active surface area, and higher oxide content which can lead to sub-surface oxygen.^45^ The sub-surface oxygen can stabilize the intermediates and thereby facilitating the *CO–CO* coupling. It was also noted that co-deposition of metal oxide and GO yields a better catalyst combination whether one uses CFP (fCu/Cu_2_O-GO) or Pt plate (pCu/Cu_2_O-GO) as a cathode during their electrodeposition as compared to the electrodes obtained in a layer-over-layer manner (both, physical layering of metal oxide nanostructures on GO (pCu/Cu_2_O@GO) and electrodeposited metal oxide nanostructures on physically adsorbed GO layer (fCu/Cu_2_O@GO)) on CFP. A layer-over-layer (pCu/Cu_2_O@GO) electrode fabrication is least active for ERC than the one where metal oxide nanostructures are electrodeposited on GO, among the studied samples. This may be due to inefficient interaction of GO with the metal oxide nanostructures in case of layer-over-layer electrodes which is explicitly confirmed from the Raman spectral characterization. Thus, the combination of the physico–chemical properties of an electrodeposit *viz.* surface structure (*i.e.* higher surface area), electronic interaction with the support material (as confirmed from Raman spectroscopy), and larger oxide content (as confirmed from XPS, XRD, and EDX) was found to be the most appropriate combination that show the best activity among the studied materials.

Furthermore, as ERC was carried out under reducing conditions, in order to study the effect of ERC on the crystal structure and the morphology of the catalyst, the electrode catalyst materials were removed from the electrode surface and characterized using XRD and SEM imaging. The ratio of Cu_*x*_O/Cu phase calculated from XRD overlays before and after the ERC (Fig. SI-11 is tabulated in Table SI-1†) which shows that the oxide content decrease after post electrolysis which suggests that oxide phase is not highly stable under the high applied negative potentials. We found that there is decrease in the ratio of Cu_*x*_O/Cu in all samples as summarized in Table SI-1.† It is important to mention here that the literature related to the role of oxide layers/contents in Cu-based catalysts for ERC suggest that the residual oxides are unstable in the oxide-derived Cu catalysts during ERC and where most of the copper oxides are reduced to Cu or Cu^+^ species however a small quantity of the original oxide content remain in the sample even after ERC. For example, Lee *et al.*^44^ and Mistry *et al.*^45^ claimed that residual oxides are unstable in the oxide-derived Cu catalysts during ECR where the residual oxide content analyzed *ex situ* with secondary ion-mass spectrometry (SIMS) was found to be only <1% of the original oxide content remained in the samples. Similarly, Eilert *et al.*^46^ on the basis of both ambient pressure X-ray photoelectron spectrum (APXPS) and quasi *in situ* oxygen K-edge electron energy-loss spectra (EELS) prove the absence of residual copper oxide in the reduced electro-catalyst. Presence of oxides, along with metallic structures, offer sites with multiple vacancies and increases the catalytic activity of the nanostructures 40. Similar results are noted in our study as well, where the oxide content in the copper oxide electro-catalysts as determined from the XRD measurements carried out on these samples post-electrolysis was found to decrease after ERC (noted from the decrease in relative peak intensities of copper oxide to metallic copper in the XRD patterns).

Overall, we can say that the enhanced activity of composites is also assisted by the sub-surface oxides that are remnant after ERC as suggested by Lee *et al.*^44^ and Mistry *et al.*^45^ Also, GO provided a scaffold which increased the stability of the catalyst as can be inferred from FESEM images of spent catalysts (Fig. SI-12†) where catalysts in composite with GO are not morphologically changed. This could be because of potential of GO to change electronic structure of the interacting metal oxide nanostructures with it and acting as a scaffold and support for the active metal catalysts.^48^Table 1 summarizes the performance comparison of the present catalyst with the results from a few other recent copper/copper oxide based electro-catalysts for ethylene production from electrochemical reduction of CO_2_. The comparison of the F.E. values and the mass activity obtained at a respective applied potential suggests that the Cu/Cu_*x*_O-based electro-catalyst presented in our study shows reasonably higher mass activity (194 mmol g^−1^ h^−1^) and comparable F.E. value of 34% at comparatively lower applied potential of −0.98 V *vs.* RHE.

**Table 1:** Comparative literature reports on different state of art copper based electrocatalysts for production of ethylene from CO_2_. Our finding has also been included for comparison

S. no.	Electrocatalyst	Applied potential	F.E._C_2_H__4___ (%)	Mass activity	Reference
1	Cu_2_O/NRGO	−1.40 V (RHE)	19.7	136.1 mmol g^−1^ h^−1^	49
2	Cu_2_O/ILGS	−1.30 V (RHE)	14.8	*—*	50
3	Cl derived Cu	−1.06 V (RHE)	37.0	—	51
4	Cu on pyridinic-N rich graphene oxide	−0.90 V (RHE)	19.0	—	52
5	Cu/Cu_2_O	−1.10 V (RHE)	36.3	—	53
**6**	**Cu/Cu**_***x***_ **O-GO**	**−0.98 V (RHE)**	**34.0**	**194 mmol g** ^**−1**^ **h** ^**−1**^	**Present work**

In conclusion, we precisely, for the present study, succeeded in crafting a pCu/Cu_*x*_O-GO composite through a simple, high yielding, and economic electro-deposition approach. The resulting catalysts exhibit a higher faradaic efficiency that too with lower potentials for electrochemical reduction of CO_2_ to C_2_H_4_. In general, it has been found that the manifestation of several parameters including oxide contents in an electrocatalyst, *I*_D_/*I*_G_ ratio due to GO support and mode of fabrication of working electrodes along with porous microstructures of the nano-composite contributes crucially towards CO_2_ electro-reduction. This study corroborates the synergism exhibited by copper oxide and GO for enhanced ERC and promise as excellent catalysts for the same. These findings can serve for preparation of promising ERC catalysts by tuning specific physical and chemical properties.

## Conflicts of interest

There are no conflicts to declare.

## Supplementary Material

RA-010-D0RA02754E-s001
