# Supplementary material for: Electrochemical reduction of CO2 to ethylene on Cu/CuxO-GO composites in aqueous solution
Source: RSC Adv. 2020 May 6;10(30):17572–81. doi: 10.1039/d0ra02754e (PMC9053623; doi:10.1039/d0ra02754e)
Supplement: RA-010-D0RA02754E-s001 [file RA-010-D0RA02754E-s001.pdf]

Electronic Supplementary Material (ESI) for RSC Advances.
This journal is © The Royal Society of Chemistry 2020

SUPPLEMENTARY INFORMATION

Electrochemical Reduction of CO_2 to Ethylene on Cu/Cu_xO.GO composites in Aqueous Solution

Nusrat Rashid[1], Mohsin Ahmad Bhat[2], U K Goutam[3], Pravin P. Ingole*[1]
[1]Department of Chemistry, Indian Institute of Technology Delhi, New Delhi, India 110016.
[2]Department of Chemistry, University of Kashmir, Srinagar, Jammu & Kashmir, India.

The role of GO in the electrolyte on the synthesis of composites bath was accounted for by taking EIS of the electrolyte bath with and without GO and with Pt or CFP as working electrode.

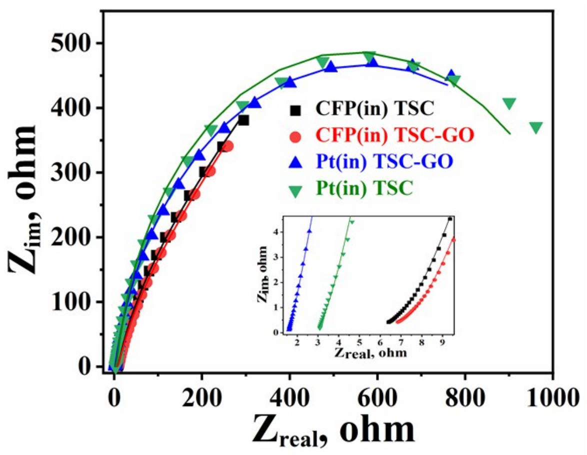

Figure SI-1: EIS of Pt and CFP in a 0.2 M TSC solution with or without GO. Inset showing the change in the solution resistance. The graphs were fitted with [R_s([RQ]Q) W] circuit.

Material Characterization:

The XRD patterns of pCu/Cu_xO and pCu/Cu_xO-GO (figure SI-2 (b)) depicts major diffraction peaks positioned at 43° and 51° corresponding to (111) and (200) planes of *fcc* structure of metallic copper (JCPDS No. 85-1326). Few minor peaks located at 36° and 42° related to diffraction from (111) and (200) planes of *fcc* Cu_2O (JCPDS No. 05-0667) can also be noticed in these XRD patterns. Interestingly, upon addition of GO in case of pCu/Cu_xO-GO, comparatively more oxide phase of copper was observed than pCu/Cu_xO was noticed. The XRD pattern of pCu/Cu_xO-GO depicts diffraction peaks positioned at 36° and 42° related to reflections from (111) and (200) planes of Cu_2O phase. Besides, few peaks located at 35°, 38°, and 49° corresponding to diffraction from (11$\bar{1}$), (111), and (200) planes of *monoclinic* CuO (JCPDS file No. 48-1548), few significant peaks from metallic Cu are also noted. Thus, the presence of GO in the electrophoretic setup seems to favour the oxide phase than the metallic Cu in pCu/Cu_xO-GO which is a manifestation of oxygen group transfer from GO to copper occurring when copper interacts with the available -C-O bonds of GO. This oxide phase preference can be realised from XRD peak ratio of Cu_xO/Cu as given in table SI-1; composites have more intense peaks for Cu_xO than Cu.

The XRD patterns for fCu/Cu_xO, fCu/Cu_xO-GO, and fCu/Cu_xO@GO (figure SI-2 (c)) show the combination of different copper phases and again addition of GO leads to more oxide phases as inferred from pCu/Cu_xO and pCu/Cu_xO-GO (table SI-1). However, the change in

cathode material from Pt to CFP does not appreciably change the crystallographic phases obtained through electrochemical deposition though morphological changes are observed from FESEM images. These observations from the XRD project the influence of GO on phase preference of copper/copper oxide nano-structures which we attribute to the interaction between GO and copper, where the former one is reduced and the later adheres to GO as oxide. This variation in crystalline phases would directly influence the electrocatalytic activity of these copper-based electro-catalyst materials.

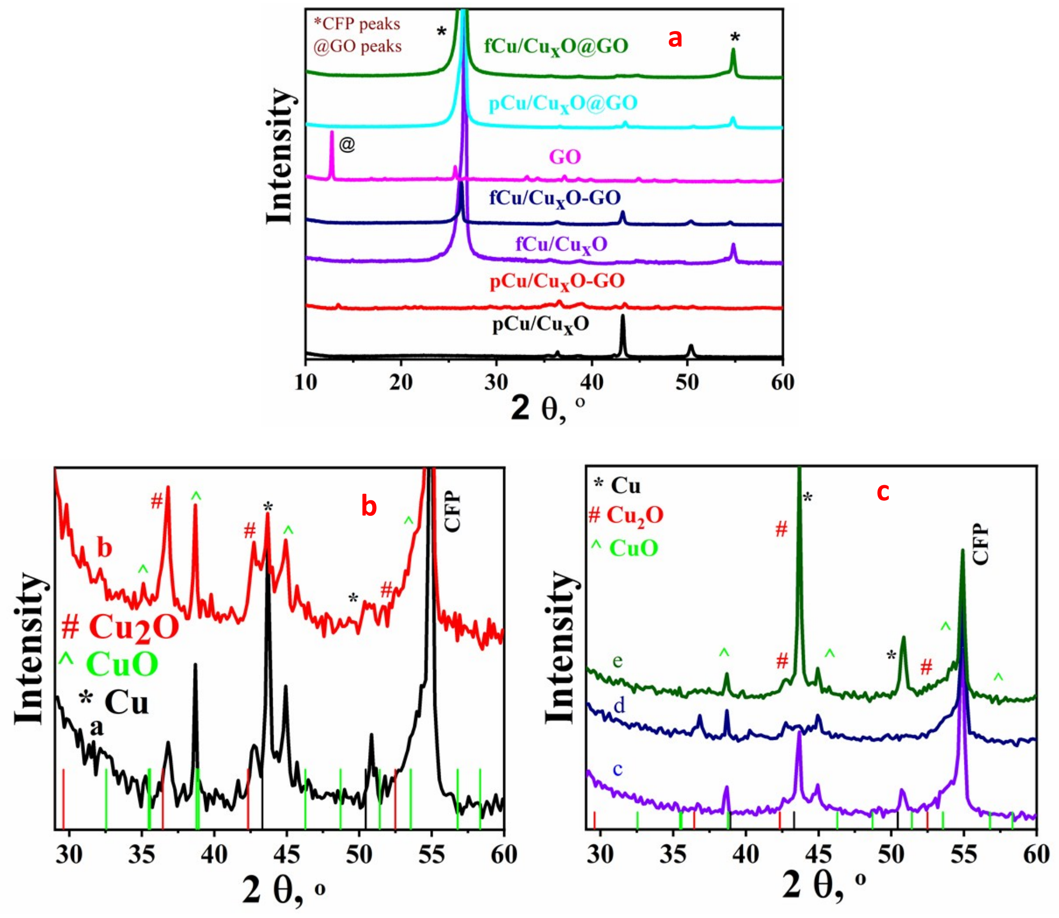

SI-2. *XRD patterns of Cu/Cu_xO and its composites with GO. (a) overlay of Cu/Cu_xO-GO composites with intense CFP peaks; (b) (a trace) pCu/Cu_xO, and (b trace) pCu/Cu_xO-GO; (c); (c trace) fCu/Cu_xO, (d trace) fCu/Cu_xO-GO, (e) fCu/Cu_xO@GO. The bar diagram in both the panels corresponds to JCPDS data from file numbers* 48-1548 *of fcc-CuO (green bars),* 05-0667 *of fcc-Cu_2O (red bars), and* 85-1326 *of fcc-metallic Cu (black bars).*

Further to support the formation of a composite and deduce an idea about the extent of reduction of GO into rGO (reduced graphene oxide because of composite formation) (which also reflects the extent of defects in GO sheets manifested in I_D/I_G ratio) hence a presentation of extent of metal – GO interactions. Raman spectra were recorded for all the composite samples containing GO, as shown in the SI-3. The characteristic peaks were observed for pristine GO sample i.e. peaks positioned at 1350 cm^{-1} corresponding to D band (related to degree of order/disorder due to a breathing k-point phonon of A_1g symmetry) and at 1590 cm^{-1} corresponding to G band (related to the E_2g phonon of sp^2 hybrid carbon atoms, which is an

indicator of the stacking structure) [1]. All the composite samples demonstrate an enhancement in intensity of D band and lowering in intensity of G band as compared to pristine GO indicating the formation of a composite between Cu/Cu_xO and GO. Furthermore, ratio of intensities of D band to that of G band (i.e. I_D/I_G ratios) estimated from the Raman spectra (table SI-1) suggest a trend which is in accordance with the observations made from XRD and EDX analysis. Particularly, electrodeposited sample using Pt cathode i.e. pCu/Cu_xO-GO shows highest I_D/I_G intensity ratio of 1.23, followed by fCu/Cu_xO@GO with I_D/I_G = 1.19, fCu/Cu_xO-GO with I_D/I_G = 1.05, pCu/Cu_xO@GO with I_D/I_G = 0.92, and pristine GO showing the lowest I_D/I_G ratio of 0.88. Pertinent to mention that higher I_D/I_G ratio reflects a greater extent of charge shared between metal and the GO support.

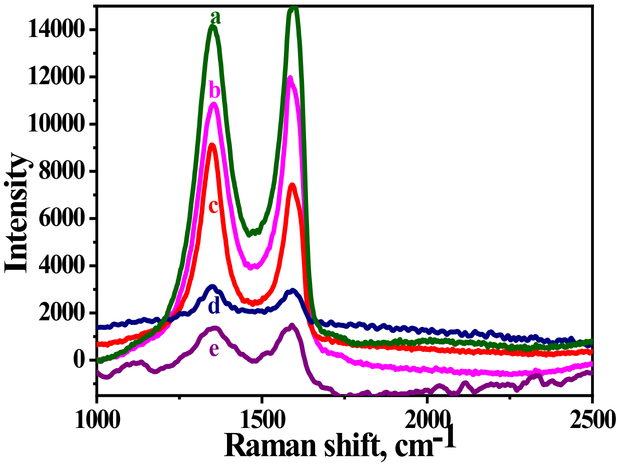

Figure SI-3. *Raman spectra of the composites with D and G bands at 1350 cm^{-1} and 1590 cm^{-}, (a) fCu/Cu_xO@GO, (b) GO, (c) pCu/Cu_xO-GO, (d) fCu/Cu_xO-GO and (e) pCu/Cu_xO@GO.*

Table SI-1: Summary of the physico-chemical parameters obtained from XRD and Raman spectral analysis.

Samples	GO support	Cathode (synthesis)	I_D/I_G ratio	Cu_xO/Cu before electrolysis	Cu_xO/Cu after electrolysis
pCu/Cu_xO	--	Pt	--	0.890	0.593
pCu/Cu_xO-GO	GO	Pt	1.31	2.60	0.82
fCu/Cu_xO	--	CFP	--	1.17	0.977
fCu/Cu_xO-GO	GO	CFP	1.01	2.02	1.263
fCu/Cu_xO@GO	GO	CFP	0.93	0.851	0.536
pCu/Cu_xO@GO	GO	--	--	--	--
GO	GO	--	0.92	--	--

SI-4: Elemental mapping of different samples.

All samples were found devoid of any impurity, and distribution of constituent elements was homogenous**.**

(a) pCu/Cu_xO

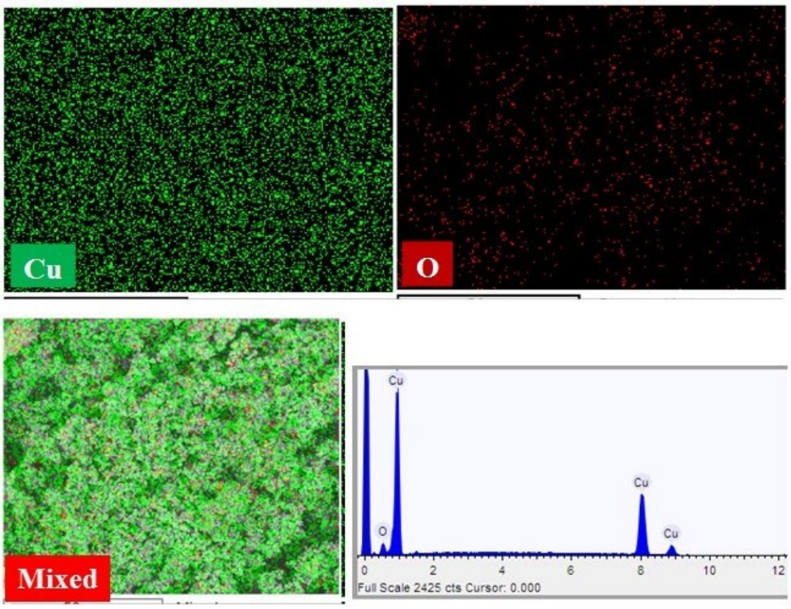

(b) pCu/Cu_xO-GO

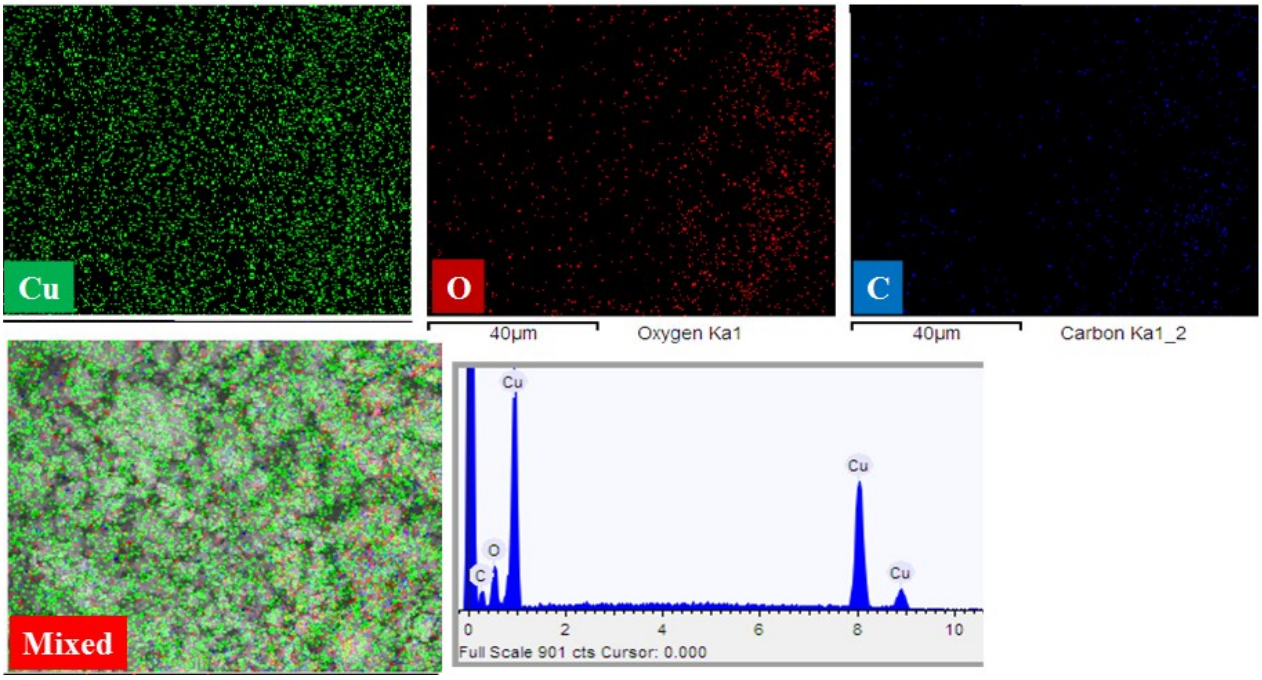

(c) fCu/Cu_xO

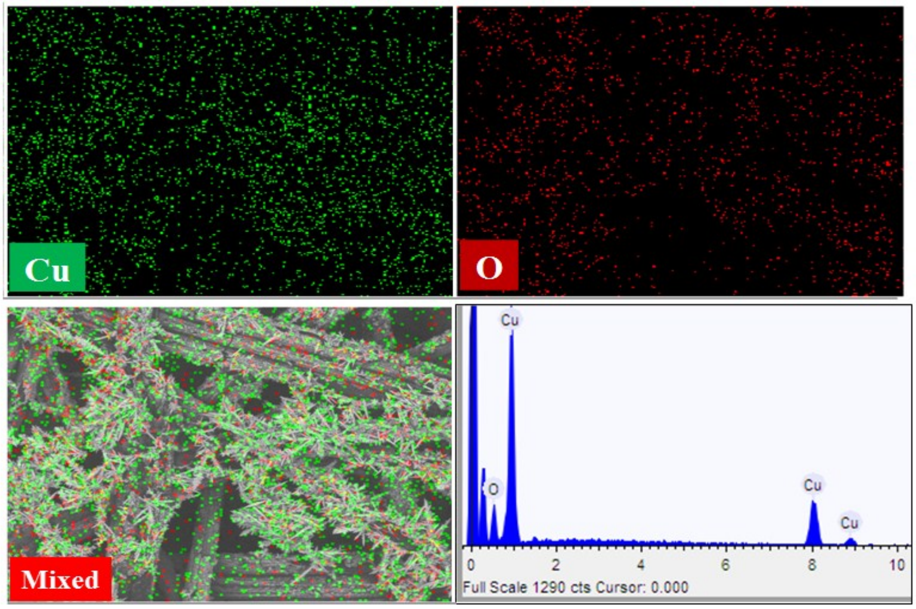

(d) fCu/Cu_xO-GO

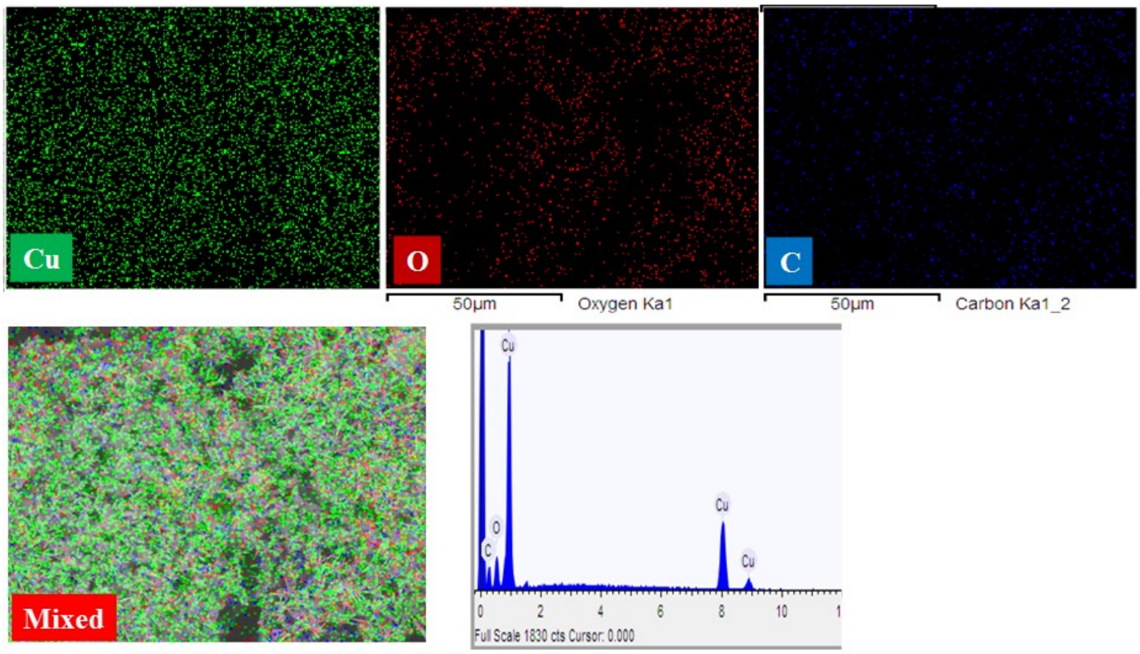

(e) fCu/Cu_xO@GO

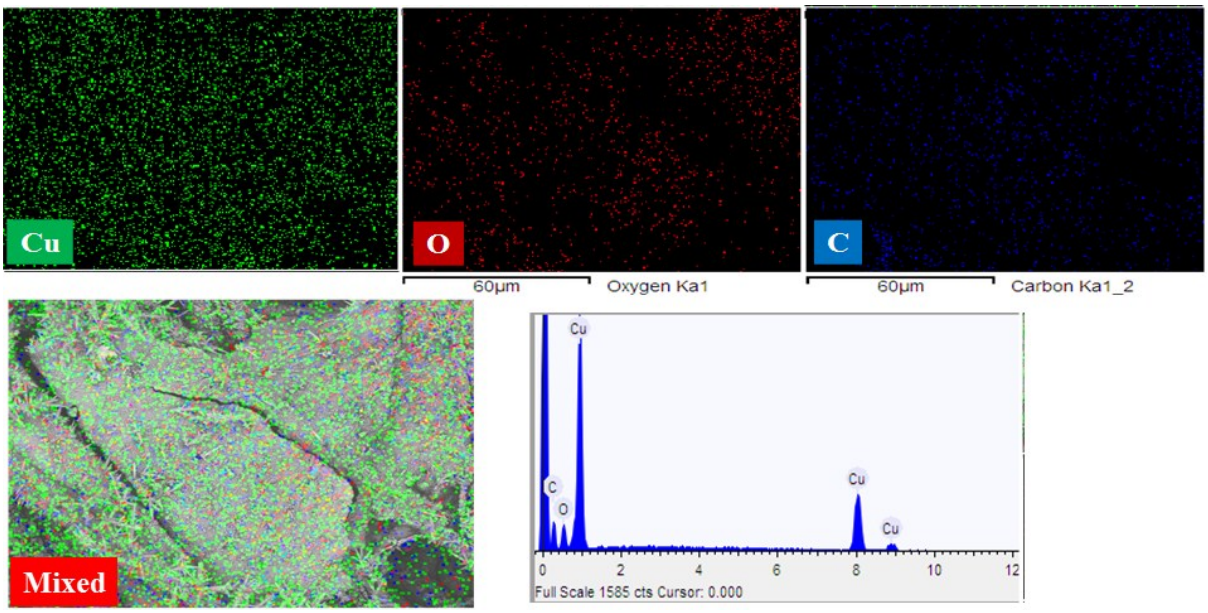

__Figure SI-4__: Detailed EDX elemental scans for the materials.

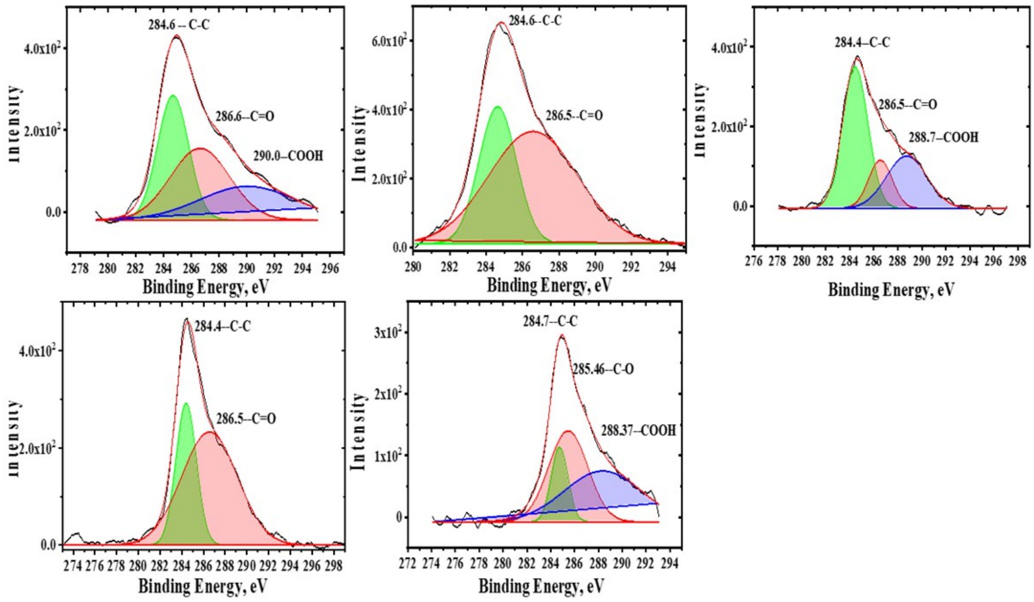

***Figure SI-5:** Detailed XPS scan for C 1s in the materials prepared, showing peaks for -C-C, -C=O and -COOH groups. Materials without GO show presence of all three functional groups while materials where GO was added show that -COOH group is no longer present suggesting locking of the group by interacting with metal centre. (a) pCu/Cu_xO, (b) pCu/Cu_xO-GO, (c) fCu/Cu_xO, (d) fCu/Cu_xO-GO, and (e) fCu/Cu_xO@GO.*

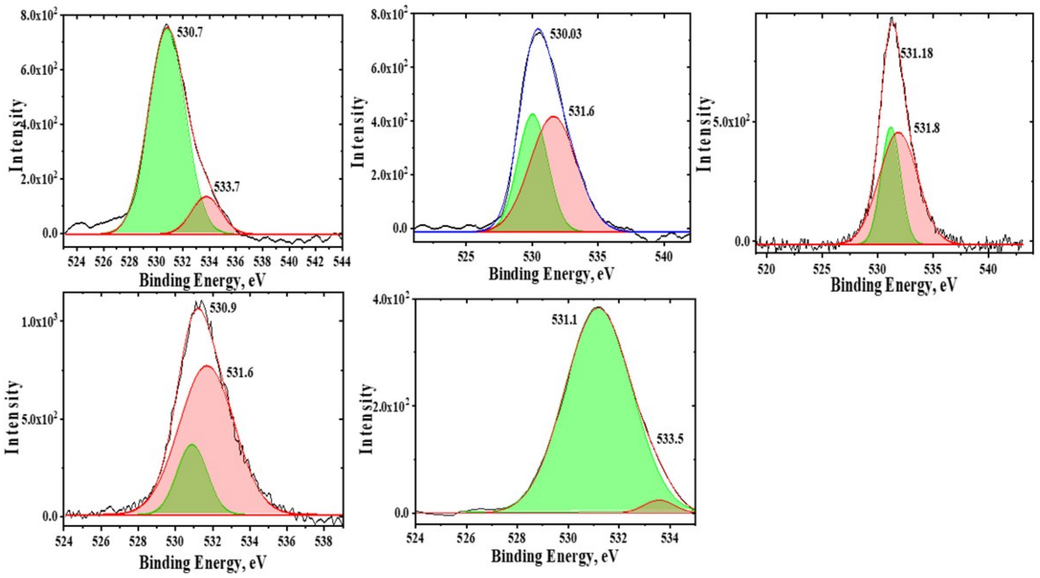

Figure SI-6: *Detailed XPS scan for O 1s in all of the materials showing peaks for oxide phase and -OH phase. (a) pCu/Cu$_x$O, (b) pCu/Cu$_x$O-GO, (c) fCu/Cu$_x$O, (d) fCu/Cu$_x$O-GO, and (e) fCu/Cu$_x$O@GO.*

Table SI-2: *XPS peak positions along with ratio of Cu^{2+} to Cu^{+}.*

S.No.	Sample	Cu 2p	O 1s	C 1s	Cu^{++}/Cu^{+} (area)
1	pCu/Cu$_x$O	932.6<br>933.8	530.7<br>533.7	284.6<br>286.6<br>290.0	1.64
2	pCu/Cu$_x$O-GO	932.5<br>933.9	530.0<br>531.6	284.6<br>286.5	3.99
3	fCu/Cu$_x$O	931.8<br>933.7	531.1<br>521.8	284.4<br>286.5<br>288.7	1.56
4	fCu/Cu$_x$O-GO	931.8<br>933.5	530.9<br>531.6	284.4<br>286.5	4.81
5	fCu/Cu$_x$O@GO	932.8<br>934.0	531.1<br>533.5	284.7<br>285.4<br>288.3	1.25

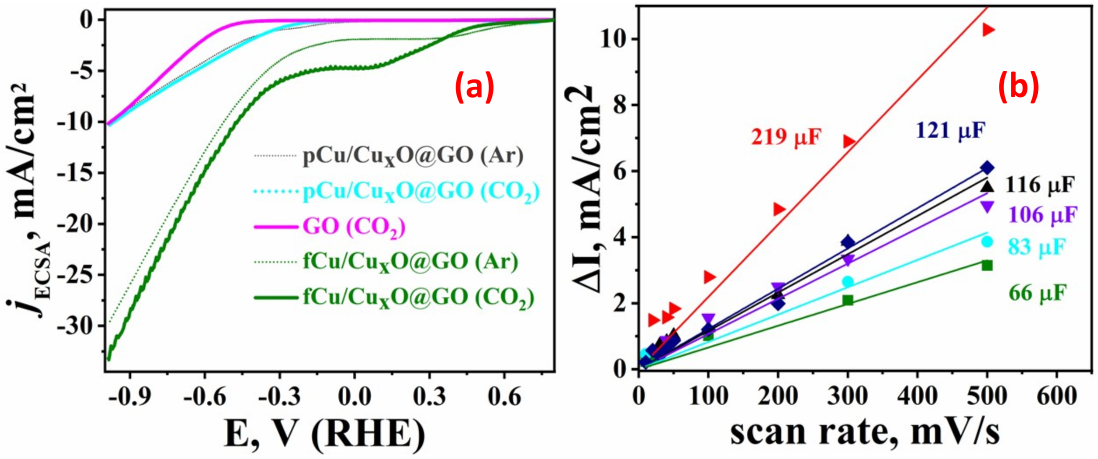

Figure SI-7: *(a) LSV traces of fCu/Cu$_x$O@GO and pCu/Cu$_x$O@GO and GO in 0.2 M $KHCO_3$ saturated with CO_2. (b) Current as a function of scan rate to calculate capacitance of the materials, ▲ pCu/Cu$_x$O, ► pCu/Cu$_x$O-GO, ▼ fCu/Cu$_x$O, ♦ fCu/Cu$_x$O-GO, ● fCu/Cu$_x$O@GO, ■ pCu/Cu$_x$O@GO.*

The only detected liquid product was formic acid (NMR) at 8.4 ppm and its accumulative concentration for one hour was highest recorded at fCu/Cu$_x$O with 8%, and it was recorded at 3.9% with pCu/Cu$_x$O-GO and not detected at electrodes modified with fCu/Cu$_x$O@GO and pCu/Cu$_x$O@GO.

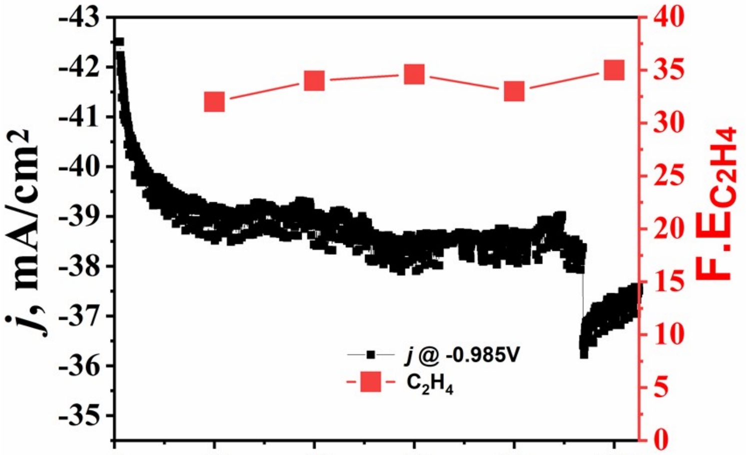

Figure SI-8*: The time-dependent current density and F.E. curves for the production of ethylene on pCu/CuxO-GO at -0.98 V vs RHE over the time span of 100 minutes.*

Electrochemical Impedance analysis (EIS): To probe the charge transfer dynamics, EIS studies (SI-9) at different potentials and in presence and absence of CO_2 were recorded. The equivalent circuits for the Nyquist plots are given in Figure SI-9. At open circuit potential in presence of argon, charge transfer resistances are more than in presence of CO_2 suggesting that reduction of CO_2 may be easy on these surfaces than HER, occurring in absence of CO_2, and pCu/Cu$_x$O-GO composites show better electrochemical activity. At the onset potential, the reaction starts and charge transfer resistances in argon saturated are lowered and suggesting

HER occurring at the surfaces. In presence of CO_2, pCu/Cu_xO-GO shows minimum resistance hence should be efficient towards the ERC; also, pCu/Cu_xO-GO is not very good for HER even in absence of CO_2.

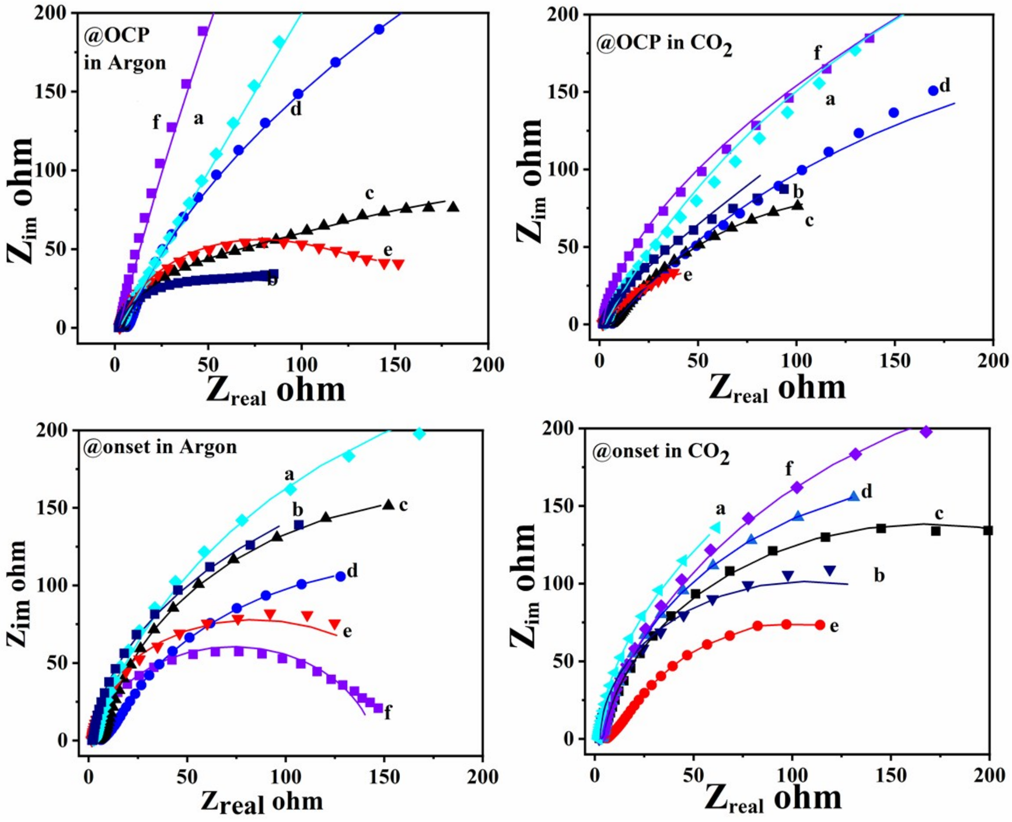

Figure SI-9: *Nyquist plots recorded in presence and absence of CO_2 at OCPs (upper panel), and onset potentials (bottom panel). (a) fCu/Cu_xO@GO, (b) fCu/Cu_xO-GO, (c) pCu/Cu_xO, (d) fCu/Cu_xO, (e) pCu/Cu_xO-GO and (f) pCu/Cu_xO@GO.*

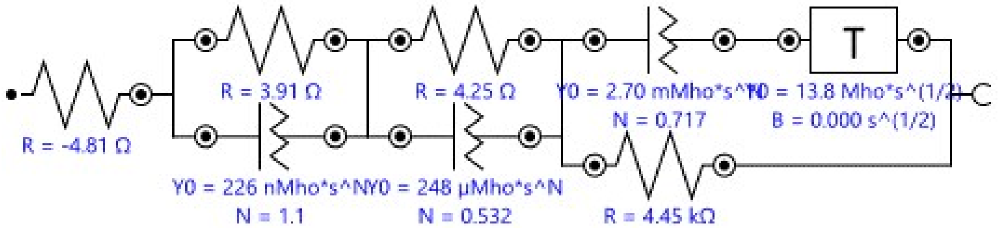

Figure SI-10 (a): *Equivalent circuit in Argon saturated solution.*

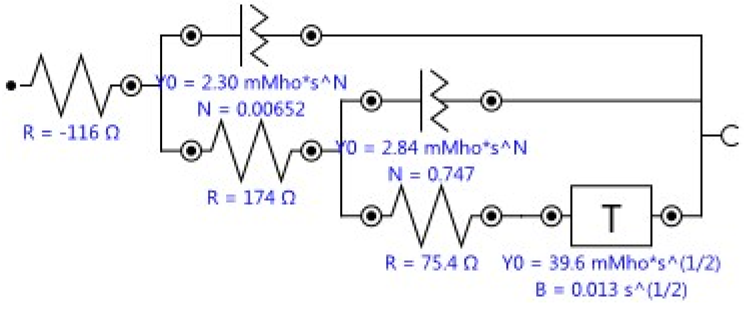

Figure SI-10 (b): *Equivalent circuit in CO_2 saturated solution.*

Stability of catalysts:

ERC at higher negative potentials is associated with reduction of the copper oxide and this reduction leads to the degradation of catalyst activity and breakdown of morphology. The nano-morphological stability of the materials after prolonged electrolysis was checked by analyzing the spent (used materials) with XRD and FSEM. Figure SI-10 shows the phase transformation of the catalysts after electrolysis of 7 hours at -0.985 V vs RHE. Peaks because of metallic copper are more intense than the copper oxide peaks which is due to the electrochemical reduction of CuxO during ERC. The morphology of the catalysts after bulk electrolysis was found to be intact as seen in FESEM images, SI-11, wherein pCu/CuxO, conical structures are not ubiquitous. The pCu/CuxO-GO shows agglomeration of particles but looks similar to the unspent assembly of particles on GO. fCu/CuxO and fCu/CuxO-GO show no pronounced effect of prolonged electrolysis, while fCu/CuxO@GO shows agglomeration of the particles over GO sheets. This morphological stability even after 7 hours of continuous electrolysis highlights the stability and durability of these synthesized catalysts.

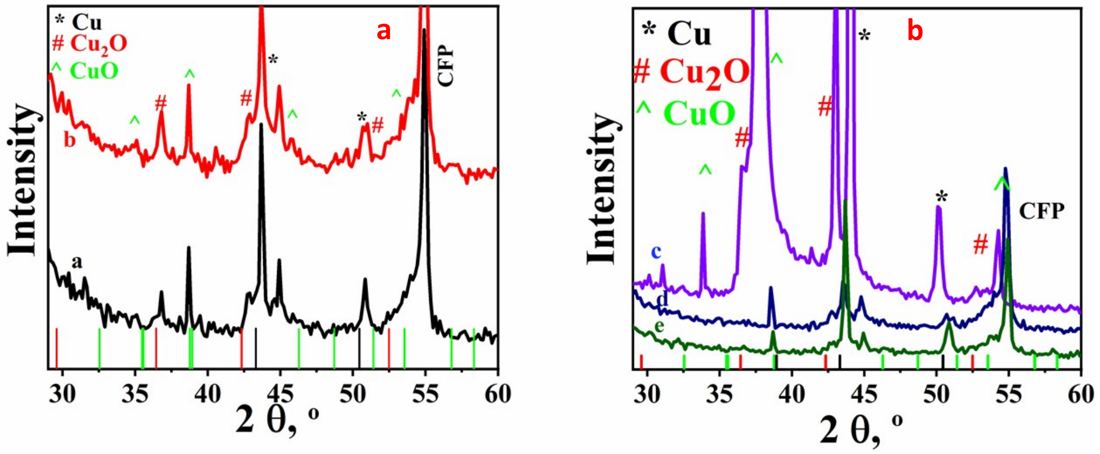

Figure SI-11: *XRD graphs of the catalyst surfaces post-electrolysis at -0.985V, (left panel) (a trace) pCu/Cu$_x$O, (b trace) pCu/Cu$_x$O-GO, and (right panel) (c trace) fCu/Cu$_x$O, (d trace) fCu/Cu$_x$O-GO, (e trace) fCu/Cu$_x$O@GO.*

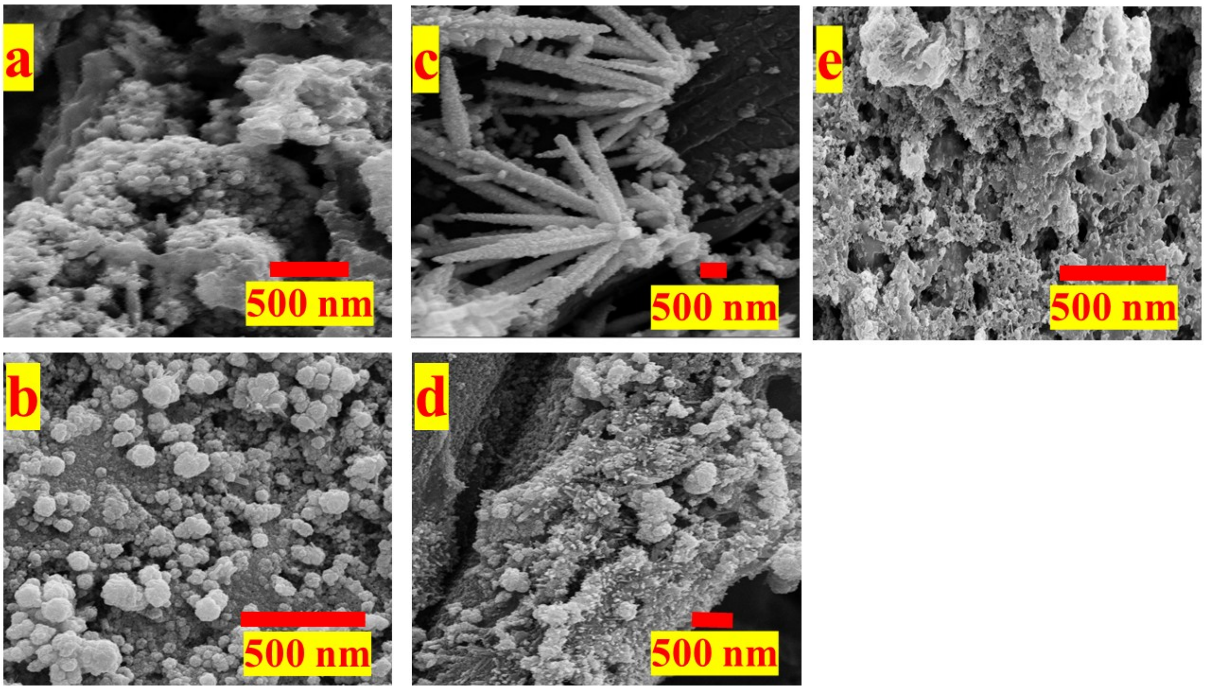

Figure SI-12: *morphological transformation of the spent materials. The images show that the prolonged electrolysis does not affect the morphology of composites and hence the catalysts are stable for longer run times for ERC, (a) pCu/Cu$_x$O, (b) pCu/Cu$_x$O-GO, (c) fCu/Cu$_x$O, (d) pCu/Cu$_x$O-GO and (f) fCu/Cu$_x$O@GO.*

References:

1. R. Saito, M. Hofmann, G. Dresselhaus, A. Jorio, M. S. Dresselhaus, *Advances in Physics,* 2011, **60.**
